# A System Identification Approach to Motion Model Based on Full-Scale Ship Maneuvering Data

**DOI:** 10.3390/s26103199

**Published:** 2026-05-19

**Authors:** Yanfei Tian, Wuliu Tian, Ke Zhang, Lin Hua, Jie Wen, Fangyang Zhu

**Affiliations:** 1 Maritime College, Beibu Gulf University, Qinzhou 535011, China; feifei_whut@163.com (Y.T.); tianwuliu@bbgu.edu.cn (W.T.); zhufangyang@bbgu.edu.cn (F.Z.); 2 China Waterborne Transport Research Institute, Beijing 100088, China; wenjie@wti.ac.cn; 3 School of Naval Architecture and Ocean, Naval University of Engineering, Wuhan 430033, China; hlby123@126.com

**Keywords:** system identification, ship motion modeling, model structure identification, model parameter estimation, full-scale maneuvering experiment

## Abstract

The paper concerns motion modeling for full-scale ships under the frame of system identification (SI) principles. Several groups of full-scale ship maneuvering experiments have been implemented to collect research data. On structure identification, as an innovation, a nonlinear integrating ship motion model is identified and established. The concerned model includes 21 parameters. Under the premise of error criterion, a batch least-squares (BLS)-based parameter estimation process is used to estimate the 21 parameters. The strategy is verified for feasibility and availability by using a pragmatic case study. The accuracy of the estimated parameter values is checked by comparing the track in simulation with the trial trajectory. Research indicates that the technical process proposed in the paper from the perspective of SI principles can be applied to the modeling of ship maneuvering motion.

## 1. Introduction

The ship motion model has a wide range of applications. The model can be used to reproduce ship trajectory and predict ship maneuverability [1]. During the ship design phase, designers rely on the maneuverability prediction of new ships to optimize hull design, maneuvering equipment selection, etc., thereby avoiding maneuvering defects after the actual ship is built. This is a prerequisite for ensuring the inherent safety of ships. The ship motion model with high accuracy is of great practical value for developing simulation systems [2]. The model is one of the core technologies of navigation simulators. Sutulo and Soares [3] have discussed a number of selected topics related to ship mathematical maneuvering models, which are mainly for simulation purposes. With the development of intelligent ships and autonomous navigation technology, the maneuvering motion model has become the foundation for path planning and motion control algorithms, and is the core support for achieving autonomous navigation of ships. In port and waterway planning, the model can simulate the navigation posture of large ships in restricted waters, demonstrate the rationality of waterway width and terminal layout, and avoid blindness in engineering design. In addition, scenarios such as maritime accident replay and optimization of ship traffic management systems also rely on the precise calculation of this model. These applications, covering design, training, intelligent shipping, engineering construction, safety supervision, and other fields, deeply reflect the urgent need and necessity for building a ship maneuvering motion model.

Generally, a ship motion model consists of two elements: the model structure and the parameters. Thus, there are mainly two parts of work to complete a ship motion model: determining/selecting the model structure and identifying/estimating the parameters. The parts of work with ship motion modeling can be realized by using system identification (SI) technology.

SI techniques are developed in control engineering to build mathematical models for dynamical systems [4], which can make or improve the mathematical model of a system based on the experimental data [5,6]. Generally speaking, SI is a kind of modeling method [7]. SI is an effective method for ship motion modeling. Hwang [8] introduced the application of an SI technique in detail to a model dynamic ship motion system. Sutulo and Soares [3] have introduced and compared various SI techniques for the parameter estimation of ship motion modeling. With the development of experimental measurement technology, massive data can be observed and recorded, and SI shows its new vitality more and more in ship motion modeling.

This paper mainly focuses on ship motion modeling from perspective of SI- the three entities: data, model class, and the error criterion, where (1) groups of full-scale ship maneuvering experiments have been implemented to collect research data; (2) on structure identification, a nonlinear integrating ship motion model is identified and established; (3) the concerned model includes 21 parameters; (4) under the premise of error criterion, a BLS-based parameter estimation process is used to estimate the 21 parameters; (5) referring to Hwang [8], the accuracy of the identified parameters is checked by comparing the simulated motion. The subsequent contents are arranged as follows. Section 2 presents a literature review. Section 3 introduces the work flow of the paper. Section 4 describes the modeling process under the SI frame. Section 5 provides a result analysis. Section 6 presents the conclusions.

## 2. Literature Review

As mentioned above, there are mainly two parts of work needed to be carried out to model a ship’s motion: determining/selecting the model structure and identifying/estimating the parameters. The paper reviews some publications corresponding to these two tasks by using SI techniques.

### 2.1. Structure Identification

In general, there are two kinds of approaches to modeling ship motion. The first one is to build an explicit type of model, which is also known as the mechanism modeling method based on ship dynamic equations. This explicit type of model consists of two main kinds: the hydrodynamic model and the responding model. Moreover, the hydrodynamic model mainly refers to the integrating mathematical model and the separating mathematical model. The typical examples of the integrating type are the contributions by Abkowitz [9] and Norrbin [10,11]. These integrating-type models are still being paid attention to or improved upon nowadays. Zhang and Zou [12] and Yin et al. [13] consider the parameter identification of the Abkowitz-type model. Seo et al. [14] apply a four-degree-of-freedom (4DOF) maneuvering mathematical model based on Abkowitz’s model for assessing damaged ship maneuverability with initial asymmetry. Ni et al. [15] use the Abkowitz model as the horizontal maneuverability motion equations, to improve the behavioral realism of a maritime simulator. Kambali and Hu et al. [16] also consider an Abkowitz-type model, and then present a least-squares support-vector machines (LS-SVMs)-based method to obtain hydrodynamic derivatives. Nataraj [17] derives a nonlinear single-degree-of-freedom model with a cubic nonlinearity that is conditionally equivalent to the Abkowitz model. Yang et al. [18] develop a four-quadrant maneuvering model that extends the Abkowitz-type model by explicitly incorporating the propeller rotation rate as a control input. Yuan et al. [19] consider a three-degree-of-freedom (3 DOF) Abkowitz model, and then present an alpha evolution multi-output support vector regression (AE-MSVR) method to identify the parameters. Ning et al. [20] select the Norrbin nonlinear response model which can accurately describe the ship ‘s motion state, to solve the problem of a ship‘s curve trajectory-tracking control. Zhou et al. [21] select a nonlinear Norrbin ship motion model for simulation to ensure better performance. Gao et al. [22] use the Norrbin model of ship motion to carry out the simulation experiments, for further testing of the robustness of a controller. To examine the motion of a 100,000-ton ship, Li and Zhang [23] establish a comprehensive mathematics model by magnifying four hydrodynamic derivatives of the nonlinear Norrbin model by 1.2 times, taking full consideration of the influences of wind, waves, and tidal current on ship motion. Li et al. [24] implement both the nonlinear Norrbin and Nomoto models for MV “Yukun” for simulating and further assessing under normal and heavy sea conditions. Li et al. [25] investigate the relationship between the dimensionless cross-flow coefficient and the four hydrodynamic derivatives of the Norrbin model, in order to develop a simplified and highly accurate ship motion model.

Incidentally, the separating mathematical model is a kind of model proposed by the ship maneuvering Mathematical Modeling Group (MMG) from the Japan Towing Tank Conference (JTTC), so it is also called the MMG model [26]. The MMG model has been widely discussed, used, and referenced to this day [27]. The typical examples of the MMG type are the contributions by Inoue et al. [28], Fujino [29], and Yoshimura and Sakurai [30,31], and Lee et al. [32,33], etc. Yasukawa and Yoshimura [34] have given an introduction of the MMG standard method for ship maneuvering prediction, which can be referred to in detail when ship motion modeling is under consideration based on the MMG ideas. Yoshimura et al. [35] propose empirical formulas based on the MMG model that can easily estimate the hydrodynamic parameters required for maneuvering predictions when ship principal particulars such as ship length (L), breadth (B), draft (d), block coefficient (Cb), and trim (z) are given. Seo et al. [36] employ SI techniques to develop a maneuvering model for a leisure boat operating at medium-high speeds based on the MMG framework; the maneuvering model is modularized into hull and propeller-rudder components and optimized to improve the accuracy of ship motion predictions. Yang et al. [37] develop a physics-based numerical model to predict ship maneuverability in level ice by coupling the non-smooth discrete element method (NDEM) with MMG framework.

While the typical examples of the responding type are the contributions by Nomoto et al. [38,39], the responding-type model also has been widely discussed, used, and referenced to this day. Ren et al. [40] introduce an adaptive Nomoto model to deal with the path following the problem of ships. Wang et al. [41] consider a nonlinear ship response model for autonomous navigation control, and then propose a real-time parameter identification method based on a nonlinear Gaussian filtering algorithm. Li et al. [24] implement both the nonlinear Nomoto and Norrbin models for MV “Yukun” for simulating and further assessing under normal and heavy sea conditions. To simulate and control the motion of a cargo ship, Lan et al. [42] develop nonlinear Nomoto model, and then investigate the effects of least-squares (LS) and multi-innovation least-squares (MILS) methods for parameter identification. To study the nonparametric modeling and control of ship steering, Ouyang et al. [43] derive a black-box response model based on the Nomoto model. Sutulo and Soares [44] have reviewed and analyzed a family of such models starting from the well-known Nomoto equations. It is found that the Nomoto equations augmented with nonlinear static characteristics are used for simulating turning and zigzag maneuvering of a typical vessel with varying degrees of directional stability. Atasayan et al. [45] evaluate the performance of first- and second-order nonlinear Nomoto models in predicting the maneuverability of car carriers.

The second one is to obtain an implicit model (or so-called black-box model), which describes the input–output mapping characteristics of ship dynamics by employing artificial intelligence techniques [46]. Examples of the implicit models are the uses of artificial neural network (ANN) [47,48,49,50,51], support vector machine (SVM) [52,53,54], support vector regression (SVR) [55], relevance vector machine (RVM) [56], and deep neural network (DNN) [57,58,59,60,61,62].

Since the paper here temporarily does not discuss the building of an implicit motion model using artificial intelligence, only the mechanism-based modeling is considered. As mentioned above, so far there are three kinds of mechanism-based models for ship motion modeling: the integrating mathematical model, separating mathematical model (MMG model) and responding mathematical model. At present, the theoretical systems of ship motion modeling based on three kinds of mechanism modeling principles are very complete, and the design of structure adopted in each kind of modeling methods is relatively fixed. As a result, while modeling ship motion from the perspective of SI theory based on the existing structures of models, the main content or the difficulty in modeling does not point to selecting/determining the model structure, but to identifying the parameters in the model—a parameter estimation process.

### 2.2. Parameter Estimation

On one hand, it concerns the estimation or tuning of parameters in mechanistic models. When a model structure is determined or chosen from the already existing kinds, e.g., the integrating mathematical model (Abkowitz or Norrbin type), the separating mathematical model (MMG type), and the responding model (Nomoto type), the rest of the work is to determine the parameters in the model. Addressing this issue, SI techniques open new avenues to parameter identification of a ship motion model. Since the 1970s, SI techniques have been successfully applied in the study of ship motion modeling. For example, when a transfer function between single input (steering rudder) and multi-output (multiple state variables) was established, all parameters in the model of the transfer function were able to be estimated using SI. Until now, there have been many SI techniques employed for the parameter identification of ship motion models. Some examples are the ordinary least-squares (LS) algorithm [63], improved LS algorithm (e.g., the constrained least-square (CLS) method [4], the partial least-squares (PLS) regression [13], etc.), the recursive least-squares (RLS) algorithm [64], the improved RLS algorithm (e.g., the lattice recursive least-square (LRLS) algorithm [65], etc.), maximum likelihood (ML) estimation method [66], model reference method (MRA) [67,68], recursive prediction error (RPE) method [69,70], frequency spectrum analysis (FSA) method [71,72], Kalman filter (KF), and the extend Kalman filter (EKF) [4,8,73], wavelet filtering technique [74], genetic algorithm (GA) [2], particle swarm optimization (PSO) and the improved PSO algorithm [1,75,76], artificial bee colony (ABC) algorithm [77], ant colony algorithm (ACA) [78], fruit fly optimization algorithm (FOA) [79], novel bat algorithm (NBA) [80], Artificial Fish School Algorithm (AFSA) [81], support vector machines (SVMs) [16,82,83], and support vector regression (SVR) [19]. Zhu et al. [84] studied the comparison and optimization of some parameter identification algorithms for response ship motion models.

On the other hand, it concerns the updating or tuning of parameters in black-box models. When using traditional ANN to construct a black-box model, parameter updating primarily employs the error backpropagation + gradient descent (BP + GD) algorithm. When using SVM to construct a black-box model, parameter tuning primarily utilizes the classic and efficient sequential minimal optimization (SMO) algorithm. When constructing a black-box model using a DNN, due to the deep number of layers and numerous parameters, basic GD alone is no longer sufficient for parameter updating. Therefore, more advanced optimizers are employed in addition to error BP. These include stochastic gradient descent (SGD) and its variants, SGD with momentum, adaptive learning rate algorithms such as Adam (Adaptive Moment Estimation; currently the most popular), RMSprop (Root Mean Square Propagation), AdaGrad (Adaptive Gradient Algorithm), and AdamW (Adam with Weight Decay). These algorithms used for parameter updating/tuning are often seen in research of black-box ship motion modeling.

From the literature, SI-based ship maneuvering motion modeling mainly involves two tasks: model structure identification and parameter estimation. After the model structure is selected or designed, the remaining work is to estimate its parameters. Parameter estimation is essentially an optimization problem from the perspective of the error criterion in SI theory: estimations of concerned parameters are required to/aim to minimize or maximize an error criterion-based objective function, and thus to complete a ship motion model.

From the literature, in mechanistic modeling, the model structure is relatively fixed, such as the integrating type (Abkowitz and Norrbin models), the separating type (MMG), and the responding type (Nomoto model or K-T model). At this point, when using one of these kinds of models, the key lies in parameter estimation. According to the literature review, various methods and algorithms have been employed for parameter estimation and have achieved promising results. In non-mechanistic modeling, the model structure is not fixed and primarily depends on the designer’s approach, for example, a traditional artificial neural network (ANN) motion model or a modern deep neural network (DNN) motion model. Subsequently, an updating algorithm is adopted to refresh the parameters within this kind of non-mechanistic model, ultimately ensuring that the output of this non-mechanistic model closely approximates the observed data.

Based on the literature review, the following two phenomena or practical approaches can also be observed. On the one hand, mechanistic models can provide support for designing non-mechanistic models. For example, based on the expressions of mechanistic models, non-mechanistic models in the form of neural networks can be designed, and the parameters in mechanistic models can be mapped to the weight matrix and bias matrix in neural network models. On the other hand, the parameter update algorithm of non-mechanistic models can also be used for parameter identification in mechanistic models. For instance, when the parameters in mechanistic models are mapped to the weight matrix and bias matrix in neural network models, the update process of the weight matrix and bias matrix is also the optimization process of the parameters in mechanistic models. Once the update of the weight matrix and bias matrix is completed, parameter identification in mechanistic models is achieved accordingly.

The study is conducted based on the principle of the SI method, referencing existing model structures and parameter estimation algorithms, and drawing on the successful practices of previous researchers. This article is organized and conducted under the frame of three SI entities: data, model class, and error criterion. This article refers to the formula expression of the Norrbin nonlinear heading-rudder model and innovatively designs a nonlinear model structure with three degrees of freedom; the proposed model structure has an appearance style-like integrating model and has 21 parameters. Further, the classic BLS algorithm is adopted for parameter estimation. Finally, the modeling effect is verified by comparing the model output with the observed data.

## 3. Work Flow

**Figure 1:**
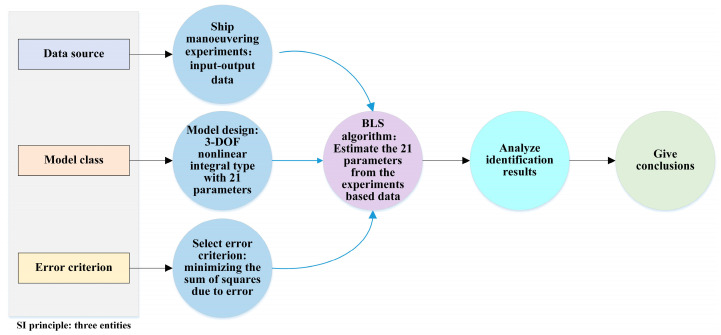
Work flow of the paper.

This article is organized and conducted under the frame of three SI entities: data, model class, and error criterion. Thus, the work flow of the paper is designed and shown in Figure 1. It includes six steps.

Step 1: Collect input–output data. Ship maneuvering experiments are executed. Input–output data of 3 DOF such as rudder, heading, planar displacement is collected, processed and visualized. Contents of this step can be found in Section 4.2.

Step 2: Design model. This article refers to the formula expression of Norrbin nonlinear heading-rudder model and innovatively designs a nonlinear model structure with 3 degrees of freedom; The proposed model structure has an appearance style like integrating model and has 21 parameters. The 21 parameters need to be estimated. Contents of this step can be found in Section 4.3.

Step 3: Select error criterion. Based on the classic and widely used least-squares (LS) concept, the sum of squares due to error (SSE) is adopted as assessment criteria. That is to say, the goal of parameter identification is to minimize SSE related with each DOF. Contents of this step can be found in Section 4.4.

Step 4: Evaluate model parameters. After the input–output data are ready, the classic and widely used BLS algorithm is utilized to estimate the 21 parameters. Contents of this step can be found in Section 4.5.

Step 5: Analyze identification results. Combining the results of parameter estimation (numerical magnitude, negative or positive), contributions to the output of the variables related to the parameters are analyzed. Observed output and the model output are compared, to reflect the degree of closeness between the model output and the observed output. In addition, some statistical indexes are calculated to the indicated test of goodness of fit of the model. This is essentially a test of the effectiveness of parameter identification. Contents of this step can be found in Section 5.

Step 6: Give conclusions. Summarize the work carried out throughout the study. Provide conclusions on feasibility of both the modeling method and parameter identification process, as well as the effectiveness of parameter identification. Shortcomings of the current research and areas for future improvement are introduced. Contents of this step can be found in the final section of the paper.

## 4. SI-Based Modeling

The mentioned references concerned with the parameter identification of the ship motion model carry out their study under three entities: the data, the set of models, and the error criterion. Thus, items in this section are arranged around the three entities. First, it gives definitions of coordinate systems and variables for indicating ship motion (see Section 4.1). Second, it declares how the specific details of the three entities are prepared, e.g., as the foundation of research, the data collection, and preprocessing based on ship maneuvering experiment (see Section 4.2), the design of the model and determination of parameters (see Section 4.3), and the error criterion based on the least-squares (LS) principle (see Section 4.4). Third, it introduces a BLS-based parameter identification process (see Section 4.5).

### 4.1. Coordinate Systems and Variables

**Figure 2:**
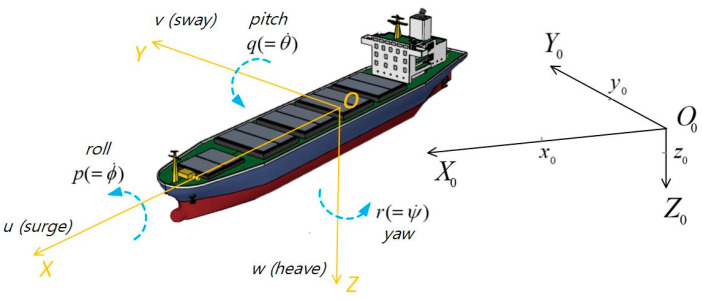
Coordinate systems and the corresponding variables for 6 DOF motion.

In the field of ship motion modeling, an earth fixed coordinate system, a body (the ship) fixed coordinate system, as well as corresponding variables are usually used to describe the ship’s six degrees of freedom (6 DOF) of motion, as shown in Figure 2.

Where the frame $O_{0}-X_{0}Y_{0}Z_{0}$ refers to the earth fixed coordinate system while the frame O−XYZ refers to the body fixed coordinate system. $O_{0}$ is a selected original point on the earth’s surface, it is usually the original point situated on where the ship starts; $O_{0}X_{0}$, $O_{0}Y_{0}$, $O_{0}Z_{0}$ respectively point to north, east, and the earth’s core. O is the original point of the O−XYZ system, it is usually situated on the ship’s center of gravity, and OX, OY, OZ respectively point to the bow, starboard, and the keel of the ship. Referring to $O_{0}-X_{0}Y_{0}Z_{0}$, $x_{o}$, $y_{o}$, $Z_{o}$ respectively are the coordinates corresponding to the three axes: ψ, ϕ, θ respectively are heading angle (also known as course), rolling angle, pitching angle; these three are called Euler angle. Referring to O−XYZ, the unwritten symbols u, v, w respectively are the ship’s surge velocity along OX, sway velocity along OY and heave velocity along OZ; The symbols p, q, r respectively are the ship’s rolling rate around OX, pitching rate around OY, and yaw rate around OZ. Specific meanings of the symbols also can be found in [10,11] and the other listed references.

### 4.2. Data Source

It is an indispensable basic work to obtain observational data of the research object through the appropriate practical physical experiment. As the foundation, several groups of ship maneuvering experiments have been executed to support the research.

#### 4.2.1. Ship in Trail Test

**Figure 3:**
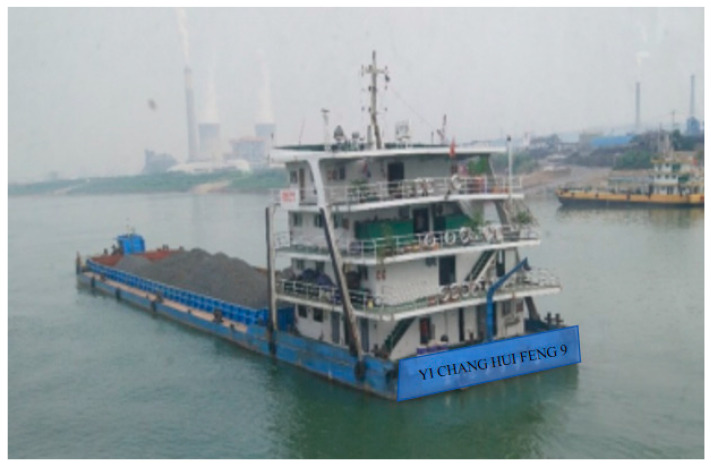
The target ship: YI CHANG HUI FENG 9.

The target ship is named “YI CHANG HUI FENG 9”, whose appearance and main hull details are present in Figure 3 and Table 1.

#### 4.2.2. Experiment Environment

The experiment environment includes date: 21 September 2016; place: Gulaobei channel, Yangtze River; air temperature: 22 °C; wind condition: there is nearly none; visibility: 10 km; flow speed: about 1.46 m/s.

#### 4.2.3. Equipment and Methods for Data Collection

Data collection mainly refers to obtaining the input and output data of the ship dynamic system. This section introduces the used equipment and methods for data collection.

**(1) System output**

System output refers to state variables of ship motion, such as the location and velocity in the $O_{0}-X_{0}Y_{0}Z_{0}$ frame, the attitude angle, and angular velocity referring to O-XYZ frame, etc.

**Figure 4:**
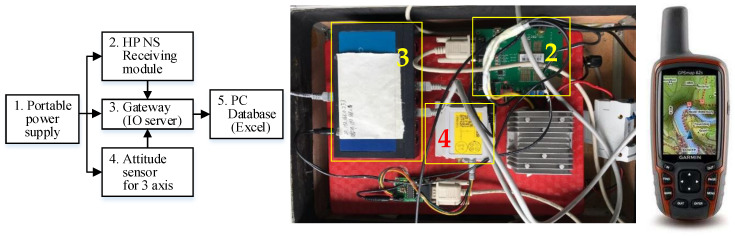
The DCS and handheld GPS for data collection.

An own-designed data collection system (DCS), as well as a handheld GARMIN GPS are used to record these state variables. The conceptual structure of DCS, physical hardwire connection of DCS, and handheld GARMIN GPS are shown from left to right in Figure 4. Briefly, the DCS uses a high-precision navigation satellite-receiving module to obtain the ship’s location, and an attitude sensor embedded in an Inertial Navigation System (INS) to sense the ship’s attitude related to three axes. The handheld GARMIN GPS is used in the bridge to continuously record the ship location automatically. Key positions are marked in this GPS equipment occasionally and manually for comparison purposes if necessary. This equipment and methods used for data collection can be seen as reported in a previous publication [85].

**(2) System input**

System input mainly includes rudder orders and engine speed by revolutions per minute (RPM) is recorded on the bridge. From an implementation perspective, videoing is used to record these maneuvering orders. The ship is navigated on a fixed RPM, but the steering rudder angles are recorded at a fixed time interval during the experiment.

#### 4.2.4. Data Processing

Essentially, with regard to a state variable, the continuous observation h at a fixed interval (1 s) is used for parameter identification in this study. However, data recorded by DCS may be interrupted or have obvious outliers. In this case, suppose that the valid data recorded by DCS from $t_{0}$ to $t_{f}$ (unit: s) are $h_{\mathrm{DCS}}$. In case of the observation at time k is missing or abnormal, a cubic spline interpolation method is used to obtain an estimation $\hat{h}\left(k\right)$. This $\hat{h}\left(k\right)$ is now treated as measurement at k and used to fill in missing data or replace abnormal data. Thus, complete data for parameter identification are achieved. This data processing method can be expressed by

(1) $$h=\left\{h_{\mathrm{DCS}}\cup \hat{h}(k)\right\},$$

At this point, the complete observation data h obtained by using Equation (2) are used for parameter identification. Then the change rate is calculated by Equation (2) since the time interval is set as 1 s.

(2) $$\dot{h}\left(k\right)=h\left(k+1\right)-h\left(k\right)$$

#### 4.2.5. Data Collection and Display

**Figure 5:**
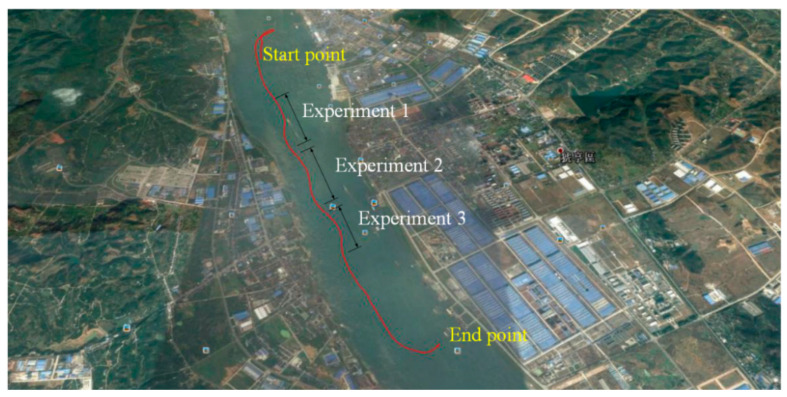
Ship track by recorded data (using the handheld GARMIN GPS).

Uploading the position data (recorded by GARMIN GPS) to Google Earth, the ship track of the whole journey during the experiment is shown in Figure 5 with the red solid line.

During the whole journey of the experiment, there are three periods when the system input (the rudder order) was relatively well observed and recorded. These periods are marked in Figure 4. System input/output data during those three periods are respectively presented in Figure 5. These data are used in this research.

**Figure 6:**
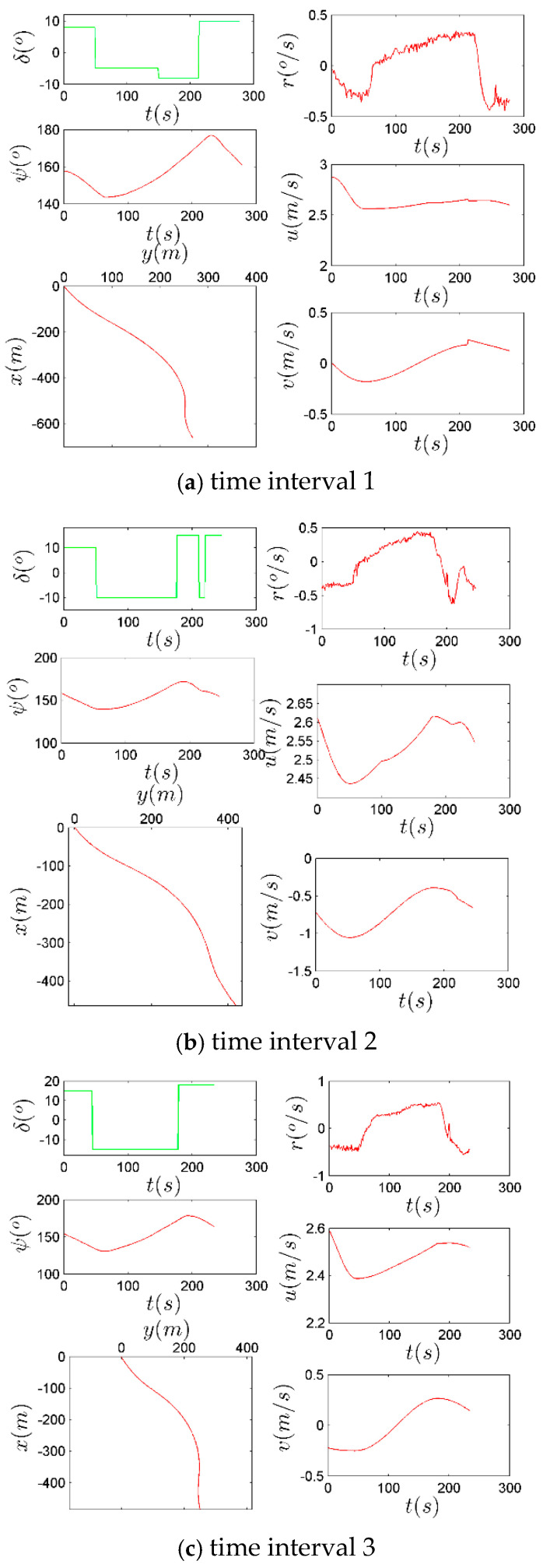
Measurement data: input and output.

After data collection and processing, the measurements (δ and #(k)) are shown in Figure 6. For the rudder angle in this figure, “+” represents the rudder which is steered to the port side while “−” is steered to the starboard side.

### 4.3. Model Structure

Considering planar 3 DOF motion, a responding ship motion model [86] displays the chain reaction of rudder angle-turning speed course ($\delta \to \dot{\psi }\to \psi$) [86]. This kind of model mainly describes the ship course’s response to the steering rudder in the plane. For example, an available structure of the second order nonlinear responding model is

(3) $$T_{1}T_{2}\ddot{r}+(T_{1}+T_{2})\dot{r}+r+\alpha r^{3}=K\delta +KT_{3}\dot{\delta },$$

where K, T (and $T_{1}$, $T_{2}$, $T_{3}$) represent ship maneuverability indices; r is the angular velocity of turning ($r=\dot{\psi }$ and ψ is ship course); δ is rudder angle; α represents a nonlinear coefficient.

Moreover, the polynomial form is usually used to build an integrating-type model. For example, Norrbin [87] had proposed a nonlinear heading model

(4) $$\dot{r}=a_{0}+a_{1}r+a_{2}r^{2}+a_{3}r^{3}+a_{4}\delta ,$$

where $a_{i}$ (i=0,1,2,3,4) is the coefficient. Gavrilin and Steen [88] used this kind of equation and identified the coefficients in order to simulate ship motion.

With this guidance, the modeling in this paper is based on the chain reaction ($\delta \to \;\dot{\#}\to \#$) and mostly takes advantage of the Norrbin polynomial form for reference. Through the process of trial and error, the final considered model structure is shown by the following uniform equations:

(5) $$\left\{\begin{array}{l} \dot{r}(k)=a_{3}r^{3}(k)+a_{2}r^{2}(k)+a_{1}r(k)+a_{0}\cdot 1+a_{uv}u(k)v(k)+a_{\delta }\delta (k)+a_{\delta \delta }\delta^{2}(k)\\ \dot{u}(k)=b_{3}u^{3}(k)+b_{2}u^{2}(k)+b_{1}u(k)+b_{0}\cdot 1+b_{uv}u(k)v(k)+b_{\delta }\delta (k)+b_{\delta \delta }\delta^{2}(k)\\ \dot{v}(k)=c_{3}v^{3}(k)+c_{2}v^{2}(k)+c_{1}v(k)+c_{0}\cdot 1+c_{uv}u(k)v(k)+c_{\delta }\delta (k)+c_{\delta \delta }\delta^{2}(k) \end{array}\right.,$$

(6) $$\left\{\begin{array}{l} r(k+1)=r(k)+\dot{r}(k)\\ u(k+1)=u(k)+\dot{u}(k)\\ v(k+1)=v(k)+\dot{v}(k) \end{array}\right.,$$

(7) $$\left\{\begin{array}{l} \dot{\psi }(k)=r(k)\\ \dot{x}(k)=u(k)\cos(\psi (k))-v(k)\sin(\psi (k))\\ \dot{y}(k)=u(k)\sin(\psi (k))+v(k)\cos(\psi (k)) \end{array}\right.,$$

(8) $$\left\{\begin{array}{l} \psi (k+1)=\psi (k)+\dot{\psi }(k)\\ x(k+1)=x(k)+\dot{x}(k)\\ y(k+1)=y(k)+\dot{y}(k) \end{array}\right..$$

The parameter vector to be identified is

(9) $$A=\left[\begin{array}{c} a\\ b\\ c \end{array}\right]=\left[\begin{array}{ccccccc} a_{3} & a_{2} & a_{1} & a_{0} & a_{uv} & a_{\delta } & a_{\delta \delta }\\ b_{3} & b_{2} & b_{0} & b_{0} & b_{uv} & b_{\delta } & b_{\delta \delta }\\ c_{3} & c_{2} & c_{0} & c_{0} & c_{uv} & c_{\delta } & c_{\delta \delta } \end{array}\right].$$

In the above equations, δ(k) represents the current actual rudder angle (unit: rad).

### 4.4. Error Criterion

The concerned 3DOF integrating ship motion model directly gives three-dimensional output: yaw rate (r), surge velocity (u), and sway velocity (v), thus the following equations are employed as the function of tracking error to evaluate quality of identification results:

(10) $$\left\{\begin{array}{l} \min J_{\dot{r}(k)}=f(e_{\dot{r}(k)})\\ \min J_{\dot{u}(k)}=f(e_{\dot{u}(k)})\\ \min J_{\dot{v}(k)}=f(e_{\dot{v}(k)}) \end{array}\right.,$$

where $J_{\dot{\#}}$ refers to the evaluation index (function value), f refers to a function, $e_{\dot{\#}}$ refers to the tracking error, k refers to sampling time (unit: s). $e_{\dot{\#}}$ is calculated by Equation (11):

(11) $$\left\{\begin{array}{l} e_{\dot{r}(k)}={\dot{r}}_{M}(k)-{\dot{r}}_{P}(k)\\ e_{\dot{u}(k)}={\dot{u}}_{M}(k)-{\dot{u}}_{P}(k)\\ e_{v(k)}={\dot{v}}_{M}(k)-{\dot{v}}_{P}(k) \end{array}\right.,$$

where ${\dot{\#}}_{M}$ represents the calculated model output after identified parameters are used in the model; ${\;\dot{\#}}_{P}$ represents observed data during experiments.

Specifically, since the sum of squares due to error (SSE) is adopted as assessment criteria based on the least-squares (LS) concept, the error criterion of identification is expressed by Equation (12)

(12) $$\left\{\begin{array}{l} \min SSE_{\dot{r}(k)}=\sum_{k=1}^{m}{e_{\dot{r}(k)}}^{2}\\ \min SSE_{\dot{u}(k)}=\sum_{k=1}^{m}{e_{\dot{u}(k)}}^{2}\\ \min SSE_{\dot{v}(k)}=\sum_{k=1}^{m}{e_{\dot{v}(k)}}^{2} \end{array}\right.,$$

where m is the total times of discrete observations within the concerned time range. Obviously, m is equal to the length of ${\dot{\#}}_{p}$ since the time interval is 1 s. Taking $\dot{r}$ as an example, the function f during modeling is specifically the sum of squared errors about $\dot{r}$ ($f=\sum_{k=1}^{m}{e_{\dot{r}(k)}}^{2}$).

### 4.5. BLS-Based Parameter Estimation

Since the model is a discrete type, the parameters can be estimated by the classical BLS. For example, if there are n groups of discrete measurement (${\dot{r}}_{\left[1\times 1\right]}(k)$, $u_{\left[1\times 1\right]}(k)$, $v_{\left[1\times 1\right]}(k)$, and $\delta_{\left[1\times 1\right]}(k)$) from $t_{0}$ to $t_{f}$, the BLS equation to estimate variable a is

(13) $$\hat{a}={\left(\Phi^{T}\Phi \right)}^{-1}\Phi^{T}R,$$

where

(14) $$a={\left[\begin{array}{ccccccc} a_{3} & a_{2} & a_{1} & a_{0} & a_{uv} & a_{\delta } & a_{\delta \delta } \end{array}\right]}^{T},$$

(15) $$\Phi_{\left[n\times 7\right]}=\left[\begin{array}{c} X(1)\\ X(2)\\ \vdots \\ X(n) \end{array}\right],$$

(16) $$R_{\left[n\times 1\right]}=\left[\begin{array}{c} \dot{r}(1)\\ \dot{r}(2)\\ \vdots \\ \dot{r}(n) \end{array}\right],$$

(17) $$X_{\left[1\times 7\right]}(k)=(\begin{array}{ccccccc} r^{3}(k) & r^{2}(k) & r(k) & 1 & u(k)v(k) & \delta (k) & \delta^{2}(k) \end{array}),\;k=1,2,\ldots ,n.$$

In the above four equations, a is a vector composed of parameters; Φ is a matrix composed of discrete measurements of the variables at all moments; R is a matrix composed of rates of change at all moments; X is a matrix composed of the observed values of the variables at moment k.

## 5. Results Analysis

Using Equation (12), the parameter vector is estimated and shown in Table 2.

From Table 2, it can be seen that estimations of parameters exhibit differentiated distributions within the range from 0 to 1 or −1 to 0. Since the established model (as expressed by Equation (5)) is data-driven and possesses the nature of a black-box model, these parameters can be interpreted as coefficients (or weights) of the corresponding variables in the prediction process. That is to say, these parameters in the established model have no physical meaning, for example, they do not have the meanings like damping coefficient, attenuation coefficient, or curve slope. Their role is similar to the weight matrix and bias matrix in the black-box model of neural networks. Specifically, the magnitude of a parameter estimation directly reflects the strength of the marginal impact of the corresponding variable on model output: a larger estimation value indicates that the corresponding independent variable has a more significant driving effect on the dependent variable, and it occupies a higher proportion in the weighted sum calculation; conversely, a smaller estimation value implies that the corresponding variable has a weaker explanatory power on the final result. This numerical difference not only reveals the importance ranking of different input variables in the weighted sum systematic calculation process, but also quantifies their respective directions of influence (positive values indicate positive promotion, negative values indicate negative suppression) and magnitude. Taking $a_{2}$ as an example, the estimation of $a_{2}$ is ${\hat{a}}_{2}=8.1866\times 10^{-2}$, which is much higher than that of other parameters. It indicates that the corresponding variable $r^{2}(k)$ makes a more prominent contribution to the calculation of output r(k) compared to other variables. Moreover, the impact of $r^{2}(k)$ on r(k) is positive.

In the parameter estimation results, some estimations are displayed as 0.0000, e.g., ${\hat{a}}_{uv}$, ${\hat{a}}_{\delta \delta }$, and ${\hat{b}}_{uv}$ in Table 2. Taking $a_{uv}$ as an example, the result ${\hat{a}}_{w\nu }=0.0000$ may stem from the following two reasons. Firstly, from the perspective of statistical inference, the true value of $a_{uv}$ may indeed be 0, ${\hat{a}}_{w\nu }=0.0000$ means that the estimation is consistent with the true value. This indicates that the independent variable u(k)v(k) has no significant linear impact on the dependent variable $\dot{r}(k)$. Secondly, from the perspective of numerical calculation accuracy, due to the limited number of decimal places retained in the output results (such as only four decimal places), the actual calculated minimum value (such as 0.000012 or −0.000008) is truncated to 0.0000 after rounding. Although its true value is not absolutely zero, it approaches zero under the current accuracy. At this point, the result ${\hat{a}}_{w\nu }=0.0000$ is an approximation of the numerical representation. Regardless of the reason, a parameter estimation of 0.0000 indicates that the corresponding variable makes no contribution to calculation of output when using this data-driven model. In other words, the contribution of the corresponding variable is negligible. Taking $a_{uv}$ as an example, ${\hat{a}}_{w\nu }=0.0000$ means that the variable u(k)v(k) makes essentially no contribution to the calculation of $\dot{r}(k)$ when using the current model Equation (5).

**Figure 7:**
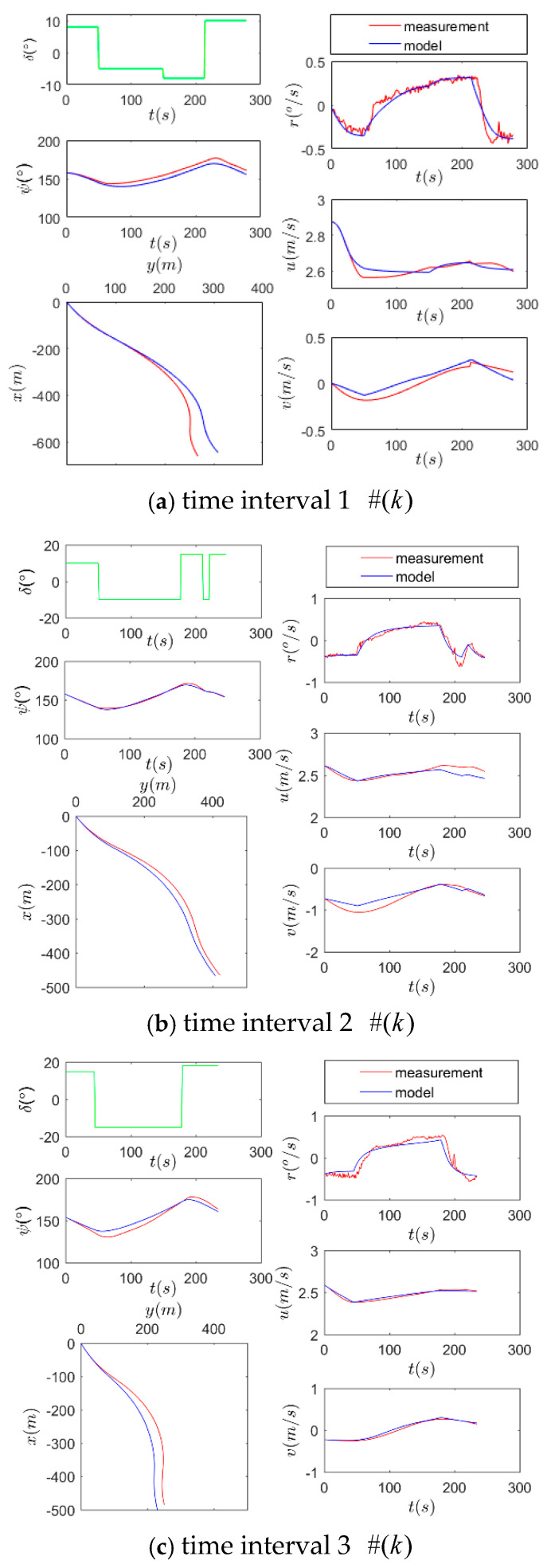
Output comparison: measurement vs. model.

Similar with Hwang [8], the accuracy of the estimated values is checked by comparing the simulated motion and the trial record. Consistently, taking the above steering commands as input, the observed output and the model output are shown in Figure 7 to make comparisons.

Figure 7a (red line) shows that from −8° to 10°, the rudder command engaged r increased to 0.31 rad/s and slowed u down by 9.2%. Figure 7b (red line) shows that from −10° to 18°, the rudder command engaged r increased to 0.38 rad/s and slowed u down by 7.2%. Figure 7c (red line) shows that from −15° to 18°, the rudder command engaged r increased to 0.61 rad/s and slowed u down by 8.1%. It can be seen in each subplot in Figure 7 that generally the trend of model output (blue line) is basically consistent with that of the observed curve (red line), although there is some deviation in local positions. This indicates that the model can describe the nonlinear characteristics of ship motion.

Moreover, the tests of goodness of fit indicated by the statistical indexes are listed in Table 3, where SSE: sum of squares due to error; SSR: sum of squares for regression; SST: sum of squares for total. CoD: coefficient of determination ($R^{2}$). For all sampling points, the final CoD ($R^{2}$) in Table 3 reaches above 0.80, indicating that the identified parameters make the mathematical model good quality.

In addition, in regard to model structure, the study found the following:

(1) Higher degree terms of a state variable (${\#}^{n}$, n≥4) contribute little on improving model output accuracy, a maximum degree of three (${\#}^{3}$) will basically meet the requirements.
(2) An addition of $\delta^{2}$ will improve model output precision, e.g., the effects mainly reflected in predictions of u and v; in general, the term with respect to $\delta^{2}$ contributes little on improving output accuracy of ψ.
(3) In general, the addition of coupling u⋅v is helpful to improve prediction accuracy of u and v, especially for v prediction; however, the coupling of u⋅v contributes little on improving the output accuracy of ψ.

## 6. Conclusions

The ship motion model is of great significance for the study of maneuverability, motion prediction, development of a simulation system, and design of a controller. The paper concerns motion modeling for full-scale ships under the frame of system identification (SI) principles. Several groups of full-scale ship maneuvering experiments have been implemented to collect research data. On structure identification, as an innovation, a nonlinear integrating ship motion model is identified and established. The concerned model includes 21 parameters. Under the premise of error criterion, a batch least-squares (BLS)-based parameter estimation process is used to estimate the 21 parameters. The strategy is verified for feasibility and availability by using a pragmatic case study. The accuracy of the estimated parameter values is checked by comparing the track in simulation with the trial trajectory. It shows that the paper proposes a uniform and concise technical process from the perspective of SI principles that can be applied to the modeling of ship maneuvering motion.

More efforts will be made in the coming studies, such as (1) on the one hand, focusing on experiment data acquisition as well as combining the existing theories and algorithms with practical engineering problems; on the other hand, paying attention to expanding application scope of the current methods to solve more problems; (2) deep research on model classes, e.g., modification of the model structure in this paper; (3) research on error criterion; the error criterion shall not only make the model fit observations well on the whole, but also keep up with the observations at feature points, so as to make the model able to fully reflect the ship maneuvering performance; in addition to SSE, other kinds of criteria can be taken into consideration for parameter identification; (4) developing some kind of automatic equipment (e.g., a software system) for the parameter identification of different types of ship motion models.

## Figures and Tables

**Table 1:** Main hull details of the target ship.

Attribute Names	Attribute Values	Attribute Names	Attribute Values
Name	YI CHANG HUI FENG 9	Identification NO.	CN20058425641
Ship type	Multi-purpose	Registration NO.	2005H4300399
Built date	8 October 2005	Limited navigation areas	Class A
LOA	76.80 m	Ship length	73.71 m
Waterline length (full)	76.41 m	Ship width	13.6 m
Molded depth	4.4 m	Maximum height	17.00 m
Draft(empty)	0.789 m	Draft(full)	3.8 m
Displacement(empty)	559.7 t	Displacement (full)	3274.55 t
Engine rated power	0.257 kw/r/min × 2	Engine reduction ratio	4:1

**Table 2:** Parameter estimation results.

Par.	Estimation						
a	−7.6957 × 10^2^	8.1866 × 10^−2^	−1.4427 × 10^−2^	−3.8415 × 10^−5^	0.0000	−1.7011 × 10^−3^	0.0000
b	2.3073	−1.8513 × 10^1^	4.9445 × 10^1^	−4.3964 × 10^1^	0.0000	−1.2880 × 10^−2^	1.6477 × 10^−1^
c	1.4926 × 10^−1^	−5.4868 × 10^−2^	−3.8912 × 10^−1^	8.7263 × 10^−4^	1.4772 × 10^−1^	−1.8430 × 10^−2^	−5.7476 × 10^−3^

**Table 3:** The test of goodness of fit.

		DOF in Earth-Fixed Frame		
		ψ	x	y
Statistical indexes	SSE	6.3267 × 10^3^	9.8708 × 10^3^	9.4442 × 10^4^
	SSR	3.1709 × 10^4^	9.3502 × 10^6^	2.6390 × 10^6^
	SST	3.8036 × 10^4^	9.3600 × 10^6^	2.7335 × 10^6^
	CoD ($R^{2}$)	0.8337	0.9989	0.9654

## Data Availability

The original contributions presented in this study are included in the article. Further inquiries can be directed to the corresponding author.

## References

[1] Dai Y., Li Y., Song J. Parameter Identification of Ship Lateral Motions Using Evolution Particle Swarm Optimization. Proceedings of the 2012 the 5th International Joint Conference on Computational Sciences and Optimization.

[2] Sutulo S., Soares C.G. (2014). An algorithm for offline identification of ship manoeuvring mathematical models from free-running tests. Ocean Eng..

[3] Sutulo S., Soares C.G. (2011). Mathematical models for simulation of manoeuvring performance of ships. Mar. Technol. Eng..

[4] Araki M., Sadat-Hosseini H., Sanada Y., Tanimoto K., Umeda N., Stern F. (2012). Estimating maneuvering coefficients using system identification methods with experimental, system-based, and CFD free-running trial data. Ocean Eng..

[5] Juang J.N. (1994). Applied System Identification.

[6] Hwang W.S., Lee D.H. (2006). System identification of structural acoustic system using the scale correction. Mech. Syst. Signal Process..

[7] Xu X.P., Wang F., Hu G. (2007). A Survey on System Identification. Mod. Electron. Tech..

[8] Hwang W.Y. (1980). Application of System Identification to Ship Maneuvering. Doctoral Dissertation.

[9] Abkowitz M.A. (1981). Measurement of hydrodynamic characteristic from ship maneuvering trials by system identification. Trans. Soc. Nav. Archit. Mar. Eng..

[10] Norrbin N.H. (1971). Theory and Observations on the Use of a Mathematical Model for Ship Manoeuvring in Deep and Confined Waters; No. SSPA-Pub-68. https://trid.trb.org/View/1951.

[11] Norrbin N.H. On the added resistance due to steering on a straight course. Proceedings of the 13th International Towing Tank Conference.

[12] Zhang X., Zou Z. (2011). Identification of Abkowitz Model for Ship Manoeuvring Motion Using ε -Support Vector Regression. J. Hydrodyn..

[13] Yin J.C., Zou Z.J., Xu F. (2015). Parametric Identification of Abkowitz Model for Ship Manoeuvring Motion by Using Partial Least Squares Regression. J. Offshore Mech. Arct. Eng..

[14] Seo J., Kim D.H., Ha J., Rhee S.H., Yoon H.K., Park J., Seok W.-C., Rhee K.P. (2020). Captive Model Tests for Assessing Maneuverability of a Damaged Surface Combatant with Initial Heel Angle. J. Ship Res..

[15] Ni S., Liu Z., Cai Y., Zhang T. (2020). A Practical Approach to Numerically Predicting a Maneuvering Vessel in Waves Oriented to Maritime Simulator. Math. Probl. Eng..

[16] Hu Y., Song L., Liu Z., Yao J. (2021). Identification of Ship Hydrodynamic Derivatives Based on LS-SVM with Wavelet Threshold Denoising. J. Mar. Sci. Eng..

[17] Kambali P.N., Nataraj C. (2023). Nonlinear dynamics of yaw motion of surface vehicles. Nonlinear Dyn..

[18] Yang Y., Moctar O.E. (2025). A four-quadrant model for ship maneuvering in forward and backward motions. Appl. Ocean Res..

[19] Yuan Q., Liu Z., Wen X., Peng J., Dong F., Zhou R., Ye J. (2025). Identification Modeling of Ship Maneuvering Motion Based on AE-MSVR. J. Mar. Sci. Eng..

[20] Ning J., Chen H., Li W., Du B. (2021). DSC-ESO Approach to Robust Sliding Model Control for Ship’s Curve Trajectory Tracking. IEEE Access.

[21] Zhou Z., Zhang Y., Wang S. (2021). A Coordination System between Decision Making and Controlling for Autonomous Collision Avoidance of Large Intelligent Ships. J. Mar. Sci. Eng..

[22] Gao S., Zhang X. (2022). Course keeping control strategy for large oil tankers based on nonlinear feedback of swish function. Ocean Eng..

[23] Li G., Zhang X. (2022). Research on the influence of wind, waves, and tidal current on ship turning ability based on Norrbin model. Ocean Eng..

[24] Li G., Zhang X. (2023). Green energy-saving robust control for ship course-keeping system based on nonlinear switching feedback. Ocean Eng..

[25] Li G., Chen Y., Yan B., Zhang X. (2024). Refinement of Norrbin Model via Correlations between Dimensionless Cross-Flow Coefficient and Hydrodynamic Derivatives. J. Mar. Sci. Eng..

[26] Zhang K., Huang L., He Y., Wang B., Chen J., Tian Y., Zhao X. (2023). A real-time multi-ship collision avoidance decision-making system for autonomous ships considering ship motion uncertainty. Ocean Eng..

[27] Zhang K., Huang L., Liu X., Chen J., Zhao X., Huang W., He Y. (2022). A Novel Decision Support Methodology for Automatic Collision Avoidance Based on Deduction of Manoeuvring Process. J. Mar. Sci. Eng..

[28] Inoue S., Hirano M., Kijima K. (1981). Hydrodynamic Derivatives on Ship Manoeuvring. Int. Shipbuild. Prog..

[29] Fujino M., Ishiguro T. (1984). A Study of the Mathematical Model Describing Manoeuvring Motions in Shallow Water Shallow water effects on rudder-effectiveness parameters. J. Soc. Nav. Archit. Jpn..

[30] Yoshimura Y. (1988). Mathematical Model for The Manoeuvring Ship Motion in Shallow Water (2nd Report): Mathematical Model at Slow Forward Speed. J. Kansai Soc. Nav. Archit..

[31] Yoshimura Y., Sakurai H. (1989). Mathematical Model for the Manoeuvring Ship Motion in Shallow Water (3rd Report): Manoeuvrability of a Twin-propeller Twin rudder ship. J. Kansai Soc. Nav. Archit. Jpn..

[32] Lee S.K., Fujino M., Fukasawa T. (1988). A Study on the Manoeuvring Mathematical Model for a Twin-Propeller Twin-Rudder Ship. J. Soc. Nav. Archit. Jpn..

[33] Lee S., Kijima K., Furukawa Y., Nakiri Y., Ibaragi H. (2005). On the Ship Manoeuvring Characteristics in Shallow Water. Trans. West-Jpn. Soc. Nav. Archit..

[34] Yasukawa H., Yoshimura Y. (2015). Introduction of MMG standard method for ship maneuvering predictions. J. Mar. Sci. Technol..

[35] Yoshimura Y., Nakamura M., Taniguchi T., Yasukawa H. (2025). Empirical formulas of hydrodynamic parameters for predicting ship maneuvering based on the MMG-model. Ocean Eng..

[36] Seo J.Y., Park H.J., Choi H., Nam B.W. (2025). Identification of a Leisure Boat’s Maneuvering Model at Medium-High Speed Using Open-Sea Trial Data. J. Offshore Mech. Arct. Eng.-Trans. ASME.

[37] Yang B.Y., Yang B.R., Song Y.L., Zhang G. (2025). Development and validation of an NDEM-based numerical model for ship maneuverability in level ice. Ocean Eng..

[38] Nomoto K., Taguchi T., Honda K., Hirano S. (1957). On the Steering Qualities of Ships. Int. Ship Build. Prog..

[39] Nomoto K., Taguchi K. (1957). On Steering Qualities of Ships (2). J. Soc. Nav. Archit. Jpn..

[40] Ren R.Y., Zou Z.J., Wang Y.D., Wang X.G. (2018). Adaptive Nomoto model used in the path following problem of ships. J. Mar. Sci. Technol..

[41] Wang S., Wang L., Im N., Zhang W., Li X. (2022). Real-time parameter identification of ship maneuvering response model based on nonlinear Gaussian Filter. Ocean Eng..

[42] Lan J., Zheng M., Chu X., Ding S. (2023). Parameter Prediction of the Non-Linear Nomoto Model for Different Ship Loading Conditions Using Support Vector Regression. J. Mar. Sci. Eng..

[43] Ouyang Z.L., Zou Z.J., Zou L. (2023). Nonparametric Modeling and Control of Ship Steering Motion Based on Local Gaussian Process Regression. J. Mar. Sci. Eng..

[44] Sutulo S., Soares C.G. (2024). Nomoto-type manoeuvring mathematical models and their applicability to simulation tasks. Ocean Eng..

[45] Atasayan E., Milanov E., Alkan A.D. (2024). A Study Evaluating the Performance of Nonlinear Nomoto Models in Predicting the Manoeuvring of Car Carrier. 2024 8th International Artificial Intelligence and Data Processing Symposium (IDAP).

[46] Luo W.L. (2016). Parameter Identifiability of Ship Manoeuvring Modeling Using System Identification. Math. Probl. Eng..

[47] Rajesh G., Bhattacharyya S.K. (2008). System identification for nonlinear maneuvering of large tankers using artificial neural network. Appl. Ocean Res..

[48] Moreira L., Soares C.G. (2012). Recursive Neural Network Model of Catamaran Manoeuvring. Int. J. Marit. Eng..

[49] Sun H.B., Shi C.J. (2014). Ship motion model identification based on Elman network. J. Shanghai Marit. Univ..

[50] An L.R., Liu M.W., Liu T., Ma J. (2022). Modeling of Ship Turning Motion Simulation Based on Bp Neural Network. J. Guangzhou Marit. Univ..

[51] Fan X., Cheng C., Hou X.R., Shi W., Gui H. (2022). Intelligent Prediction for Ship Motion Based on RBF. J. Ship Des..

[52] Luo W.L., Moreira L., Soares C.G. (2014). Manoeuvring simulation of catamaran by using implicit models based on support vector machines. Ocean Eng..

[53] Chen L., Wang J., Wang X., Wang B., Zhang H., Feng K., Wang G., Han J., Shi H. (2024). A road hypnosis identification method for drivers based on fusion of biological characteristics. Digit. Transp. Saf..

[54] Zhang Y.Y., Ouyang Z.L., Zou Z.J. (2023). Identification modeling of ship maneuvering motion in waves based on ν-support vector machine. J. Ship Mech..

[55] Lu G.Y., Yao J.X. (2021). Black-box Modeling of Ship Maneuvering by Means of SVR. Navig. China.

[56] Meng Y., Zhang X., Zhang X., Duan Y. (2025). Online identification of ship motion under different maneuvering conditions. J. Southwest Jiaotong Univ..

[57] He H., Wang Z., Zou Z., Liu Y. (2022). Nonparametric modeling of ship maneuvering motion based on self-designed fully connected neural network. Ocean Eng..

[58] Chen X., Wu P., Wu Y., Aboud L., Postolache O., Wang Z. (2025). Ship trajectory prediction via a transformer-based model by considering spatial-temporal dependency. Intell. Robot..

[59] Hao L., Han Y., Shi C., Pan Z. (2022). Recurrent neural networks for nonparametric modeling of ship maneuvering motion. Int. J. Nav. Archit. Ocean. Eng..

[60] Lou J., Wang H., Wang J., Cai Q., Yi H. (2022). Deep learning method for 3-DOF motion prediction of unmanned surface vehicles based on real sea maneuverability test. Ocean Eng..

[61] Tian Y.F., Li Z.L., Ai W.Z., Han X.H. (2024). LSTM-RNN based black-box modeling of ship manoeuvring motion. Ship Sci. Technol..

[62] Sang Y.F., Yao J.X. (2025). Research on Black Box Modeling Method of Ship Maneuvering Motion Based on Long-term and Short-term Memory Neural Network. J. Wuhan Univ. Technol. (Transp. Sci. Eng.).

[63] Zou Z.J., Wu X.H. (1985). The Parameter Identification of Non-liner K-T Equations on Ship Maneuverability. J. Wuhan Inst. Water Transp. Eng..

[64] Tzeng C.Y., Lin K.F. (2000). Adaptive ship steering autopilot design with saturating and slew rate limiting actuators. Int. J. Adapt. Control. Signal Process..

[65] Peng X.Y., Liu C.D. (2012). Extreme short time prediction of ship motion based on lattice recursive least square. J. Ship Mech..

[66] Åström K.J., Källström C.G. (1989). Identification of Ship Steering Dynamics. Automatica.

[67] Van Amerongen J. (1984). Adaptive steering of ships—A model reference approach. Automatica.

[68] Hayes M.N. (1971). Parametric Identification of Nonlinear Stochastic Systems Applied to Ocean Vehicle Dynamics. Ph.D. Thesis.

[69] Zhou W.W., Blanke M. (1989). Identification of a class of nonlinear state-space models using RPE techniques. IEEE Trans. Autom. Control..

[70] Herrero E.R., González F.J.V. (2012). Two-step identification of non-linear manoeuvring models of marine vessels. Ocean Eng..

[71] Bhattacharyya S.K., Haddara M.R. (2006). Parameter identification for nonlinear ship manoeuvring. J. Ship Res..

[72] Selvam R.P., Bhattacharyya S.K., Haddara M. (2005). A frequency domain system identification method for linear ship maneuvering. Int. Shipbuild. Prog..

[73] Reza H., Bin L., Michael B., Pan H., Meysar Z., Zheng J., Yan M., Li Y., Huang C., Ma Y. (2021). An online identification approach for a nonlinear ship motion model based on a receding horizon. Trans. Inst. Meas. Control..

[74] Zhang X., Zou Z. (2011). Application of Wavelet Denoising in the Modeling of Ship Manoeuvring Motion. J. Ship Mech..

[75] Chen Y., Song Y., Chen M. (2010). Parameters identification for ship motion model based on particle swarm optimization. Kybernetes.

[76] Wang Z., Pang C., Sui J., Zhao G., Wu W., Xu L. (2024). Time-optimal trajectory planning for a six-degree-of-freedom manipulator: A method integrating RRT and chaotic PSO. Intell. Robot..

[77] Tian Y.F., Hu S.C., Huang L.W. (2017). Off-line parameter identification of mathematical ship motion model based on artificial bee colony algorithm. Inf. Technol..

[78] Dai Y.T., Zhao X.R., Liu L.Q. (2010). Parameter Identification of Ship Longitudinal Motions Using Continuous Domains Ant Colony Algorithm. Microcomput. Inf..

[79] Wang X.G., Zou Z.J. (2012). Identification of ship manoeuvring response model based on fruit fly optimization algorithm. J. Dalian Marit. Univ..

[80] Tian Y.F., Huang L.W., Xiong Y. (2018). Parameter identification of ship motion model by using novel bat algorithm. Sci. Technol. Eng..

[81] Yang X., Shao X. (2021). Parameter Identification of Maneuvering Response Model for High-speed USV Based on AFSA. Digit. Ocean Underw. Warf..

[82] Song X., Chen Z.S. (2025). Time charter rate forecasting by Parsimonious Intelligent Support Vector regression Search Engine. Digit. Transp. Saf..

[83] Luo W.L., Soares C.G., Zou Z.J. (2016). Parameter Identification of Ship Maneuvering Model Based on Support Vector Machines and Particle Swarm Optimization. J. Offshore Mech. Arct. Eng.-Trans. ASME.

[84] Zhu M., Hahn A., Wen Y.Q., Bolles A. (2017). Comparison and Optimization of the Parameter Identification Technique for Estimating Ship Response Models. Proceedings of 2017 IEEE 3rd International Conference on Control Science and Systems Engineering.

[85] Tian Y., Huang L., Xiong Y., Chen Y. (2017). Parameter identification of an ARX type ship motion model using system identification techniques. Int. J. Smart Eng..

[86] Zhang X.K., Jia X.L., Liu C. (2004). Research on responding ship motion mathematical model. J. Dalian Marit. Univ..

[87] Norrbin N.H. (1963). On the Design and Analysis of the Zig-Zag Test on Base of Quasi-Linear Frequency Response.

[88] Gavrilin S., Steen S. (2017). Validation of ship manoeuvring models using metamodels. Appl. Ocean Res..

